# Supplementary material for: MetaRibo-Seq measures translation in microbiomes
Source: Nat Commun. 2020 Jun 29;11:3268. doi: 10.1038/s41467-020-17081-z (PMC7324362; doi:10.1038/s41467-020-17081-z)
Supplement: Supplementary file 10 — Supplementary Data 7 [file 41467_2020_17081_MOESM10_ESM.zip › File2/Confidence_High_Taxonomy/306469_out.krona.html]

Javascript must be enabled to view this page.

members
magnitude
magnitudeUnassigned
count
unassigned
taxon
rank

306469\_out

19

2
superkingdom
19

976
19
phylum

class
19
200643

171549
19
order

1
family
815

816
1
genus

821
1

SRS143876\_contig\_number\_1430
species

family
18
171552

838
genus
18

18
species

SRS013098\_contig\_number\_28803SRS013638\_contig\_number\_8956SRS014415\_contig\_number\_70SRS015590\_contig\_number\_13575SRS018888\_contig\_number\_5911SRS019285\_contig\_number\_contig-100\_9.10SRS024132\_contig\_number\_52284SRS043841\_contig\_number\_11266SRS044535\_contig\_number\_11227SRS049712\_contig\_number\_9378SRS058145\_contig\_number\_1509SRS065397\_contig\_number\_9982SRS077991\_contig\_number\_83SRS078419\_contig\_number\_10384SRS104036\_contig\_number\_1554SRS1041137\_contig\_number\_32735SRS105082\_contig\_number\_11875SRS150029\_contig\_number\_11309
165179
